# The relationship between the insulinemic potential of diet and lifestyle and risk of breast cancer: a case-control study among iranian adult women

**DOI:** 10.1186/s13690-022-01016-9

**Published:** 2023-01-09

**Authors:** Ebrahim Mokhtari, Sanaz Jamshidi, Ghazal Daftari, Hossein Farhadnejad, Farshad Teymoori, Seyed Aref Momeni, Bahram Rashidkhani, Parvin Mirmiran

**Affiliations:** 1 Student Research Committee, Nutrition and Endocrine Research Center, Research Institute for Endocrine Sciences, Shahid Beheshti University of Medical Sciences, Tehran, Iran; 2 Nutrition and Endocrine Research Center, Research Institute for Endocrine Sciences, Shahid Beheshti University of Medical Sciences, Tehran, Iran; 3 Department of Nutrition, School of Public Health, Iran University of Medical Sciences, Tehran, Iran; 4 School of Medicine, Tehran University of Medical Sciences, Tehran, Iran; 5 Department of Physical Education and Sport Science, Humanity Faculty, Semnan University, Semnan, Iran; 6 Department of Community Nutrition, Faculty of Nutrition Sciences and Food Technology, National Nutrition and Food Technology Research Institute, Shahid Beheshti University of Medical Sciences, P.O. Box: 1981619573, Tehran, Iran

**Keywords:** EDIH, ELIH, Breast cancer, Dietary pattern, Lifestyle, Women

## Abstract

**Background:**

Breast cancer (BC) is the most prevalent cancer, with a higher mortality rate in women worldwide. We aimed to investigate the association of the insulinemic potential of diet and lifestyle with the odds of BC using empirical indices, including the empirical dietary index for hyperinsulinemia (EDIH), empirical lifestyle index for hyperinsulinemia (ELIH), the empirical dietary index for insulin resistance (EDIR), and empirical lifestyle index for insulin resistance (ELIR).

**Methods:**

This hospital-based case-control study was conducted among Tehranian adult women aged≥30 years. The final analysis was performed on 134 women newly diagnosed with histologically confirmed BC as a case and 267 healthy women of the same age as control. A 168-food item food frequency questionnaire was used for assessing dietary intakes at baseline. The odds ratios (ORs) and 95% confidence intervals (CIs) of BC across tertiles of EDIH, ELIH, EDIR, and ELIR were determined using multivariable-adjusted logistic regression.

**Results:**

The mean ± SD of age and BMI of participants were 47.9±10.3 years and 29.4±5.5 kg/m2, respectively. EDIH score was related to the higher risk of BC based on fully adjusted models (OR:2.24;95%CI:1.21–4.12, P_trend_=0.016). Furthermore, subgroup analysis showed a higher BC risk with increasing EDIH score in postmenopausal women (OR:1.74, 95%CI:1.13-2.69) and those without a history of the oral contraceptive pill (OCP) use (OR:1.44;95%CI:1.02–2.04). Moreover, ELIH scores were positively associated with an increased risk of BC in postmenopausal women (OR; 1.98; 95% CI: 1.35 – 2.89), those with a family history of cancer (OR:1.94;95%CI:1.10–3.42), and in individuals who did not use OCP (OR:1.46; 95% CI:1.00–2.12).

**Conclusion:**

Our results showed a possible link between EDIH and higher BC risk. Also, higher EDIH and ELIH scores were strongly associated with a higher risk of BC in postmenopausal women, those with a family history of BC, and those who do not use OCP.

## Background

Breast cancer (BC) is the most prevalent cancer, accounting for 25% of all female-related cancers worldwide, and second cancer with the highest mortality rate in women. The disease affects about 1.4 million people annually [1]. The well-established risk factors for BC, including age, genetic mutations, premature menstruation, late pregnancy, late menopause, hormone therapy, oral contraceptives, and cancer family history, are mostly unmodifiable [2]. However, unhealthy lifestyles, including physical inactivity, obesity, and inappropriate diet, are modifiable risk factors that play an important role in cancer pathogenesis [3]. In 2018, The World Cancer Research Center reported that choosing a healthier lifestyle and changing eating habits could prevent 4 million new people from developing cancer [2].

One of the predisposing biological agents for cancer incidence and its development is insulin-related disorders, particularly hyperinsulinemia, that play a crucial role in tumor development through insulin-like growth factor-1 (IGF-1) [4]. Obese and inactive subjects are more prone to imbalanced insulin homeostasis, and unhealthy dietary patterns can lead to hyperinsulinemia and insulin resistance (IR) [5, 6].

Recently, Tabung et al. have proposed dietary and lifestyle indices to predict hyperinsulinemia and IR using serum connecting peptide (C-peptide) and Triglyceride (TGs) to high-density lipoprotein-cholesterol (HDL-C) ratio, respectively. The empirical dietary index for hyperinsulinemia and IR (EDIH and EDIR) includes only food groups related to insulin biomarker responses. In contrast, the empirical lifestyle index for hyperinsulinemia and IR (ELIH and ELIR) is composed of physical activity (PA) and body mass index (BMI), and also food groups correlated with insulin biomarkers.

Previous studies investigated the link between these insulinemic dietary and lifestyle indices and the risk of several cancer types [7–14] regarding the well-established link between insulin disorders and cancer. A recent study showed that all EDIH, EDIR, ELIH, and ELIR are positively associated with the risk of hepatocellular carcinoma (HCC) [7]. Also, two studies demonstrated that a higher EDIH score increases prostate cancer risk [8, 9]. The Wang et al. study revealed that interventions to reduce the insulinemic potential of diet and lifestyle have protective effects against digestive system cancer [10]. Furthermore, there is a significant relationship between EDIH and the higher incidence of colorectal cancer (CRC) and poorer survival in patients with CRC [11, 12]. However, Lee et al. did not observe any significant relationship between EDIH and EDIR and multiple myeloma (MM) [13].

Accordingly, most previous studies observed a positive relationship between the EDIH and various types of cancer. However, despite the benefits of ELIH and ELIR as lifestyle scores indicating collective effects of diet, PA, and BMI, few studies have examined their association with cancer risk. Also, to our knowledge, there is no study exploring these indices’ relationship with the BC risk. So, we aimed to perform a case-control study to investigate the possible association of dietary and lifestyle indices for hyperinsulinemia (EDIH, ELIH) and insulin resistance (EDIR, ELIR) with BC risk in a sample of Iranian adult women.

## Materials and method

### Study design and sample

In this hospital-based, case-control study, we recruited 136 women ≥ 30 years old and newly (<6 months) diagnosed with histologically confirmed BC at Imam Hossain and Shohada hospitals, Tehran (Iran) between September 2015 and February 2016. The control group consisted of 272 women of similar age who were admitted to the same hospital for a broad spectrum of non-neoplastic diseases unrelated to smoking, alcohol consumption, and long-term diet modification. Conditions among controls included traumas and orthopedic disorders, disk disorders, acute surgical conditions, eye, nose, ear, or skin disorders. Less than 8% of subjects approached for the interview refused to participate. Seven participants were excluded from the final analysis because their reported energy intakes were outside the ±3 standard deviation (SD) of the mean energy intakes of the population (*n*=5 controls, 2 cases). Finally, 134 cases and 267 controls remained in the final analysis.

All participants signed the informed consent, and all procedures were according to the Helsinki Declaration’s ethical standards. The ethics research committee approved the study’s protocol of the Student Research Committee, Shahid Beheshti University of Medical Sciences, Tehran, Iran.

### Dietary assessment

Participants’ dietary intake during the year before diagnosis for cases or interviews for controls was assessed in a personal interview using a valid and reliable semi-quantitative 168 food item food frequency questionnaire (FFQ) [14]. Participants were asked to specify their consumption frequency for each food item on a daily, weekly, monthly, or yearly basis. Questions on spices, including turmeric, saffron, black pepper, ginger, rosemary, and thyme, were added to the present questionnaire. Intakes were then converted to daily frequencies, and a manual for household measures was used to convert intake frequencies to daily grams of food intake [15]. The energy and nutrient content of foods was calculated by the United States Department of Agriculture (USDA) food composition table. The Iranian food composition table was used for some traditional Iranian food items that are not included in the USDA database (e.g., traditional bread). Due to Iranian regional beliefs, alcohol consumption was not asked and was unavailable for the analysis.

### Calculation Of insulinemic indices

EDIH is calculated based on two groups of food components including positive (including red and processed meat, margarine, poultry, high-energy beverages, butter, French fries, low-fat dairy, tomatoes, and eggs) and negative (coffee, high-fat dairy, green and leafy vegetables, and whole fruits.) determinants. Each of the mentioned food groups is multiplied by a particular weight previously calculated in the study conducted by Tabung and his colleague [16] and then all food scores were summed as EDIH score.

Similarly, the ELIH is determined based on a set of direct (including BMI, margarine, butter, red meat, and fruit juice) and inverse (including coffee, whole fruits, physical activity, high-fat dairy products, snacks, and salad dressing) components. Like the EDIH, the ELIH score was calculated [16].

The EDIR encompasses two groups of food components, including positive and negative determinants [16]. The positive determinants included margarine, red meat, refined grains, processed meats, tomatoes, other vegetables, fish, and fruit juice. Negative determinants included coffee, green leafy vegetables, high-fat dairy products, dark yellow vegetables, and nuts. Similarly, the ELIR is determined based on positive and negative components [16]. The positive components included were BMI, refined grains, red meat, margarine, tomatoes, fruit juice, potatoes, processed meat, other vegetables, and tea. Negative ingredients were coffee, high-fat dairy products, PA, and green leafy vegetables.

Each of the mentioned components was multiplied by a particular weight, and all weighted values were summed to form the overall scores.

### Assessment of non-dietary exposures

Trained dietitians administered all other questionnaires and measurements during the same interview. Participants’ socio-demographic, lifestyle, and clinical information collected by general questionnaires, including age (years), age at menarche (years), age at first pregnancy (years), abortion history during lifetime (yes, no), number of live births (number), breastfeeding history during lifetime (month), menopausal status at this time (pre-menopause, post-menopause), education (illiterate, less than a high school diploma, high school diploma and more), history of hormone replacement therapy during lifetime (yes, no), oral contraceptive pills(OCP) consumption history during lifetime (month), benign breast diseases history (yes, no), cancer family history (yes, no), breast cancer family history (yes, no), bra wearing (day (yes, no), night (yes, no)), marital status (single, married, divorced, widowed), smoking during lifetime (yes, no), supplement intakes in last year (including calcium, iron, zinc, selenium, B complex, Vitamin C, folic acid, vitamin A vitamin C, β carotene, vitamin E, vitamin D, multivitamins-minerals, omega-3 fatty acids, and probiotics) (yes, no; If yes, the complementary information on dose and frequency), and anti-inflammatory drug use (yes, no). Also, data on physical activity during the last year was assessed with a valid and reliable questionnaire [17].

Weight was measured to the nearest 0.5 kg using a digital scale (Seca, Germany), with the participant wearing lightweight clothing and no shoes. Height was measured to the nearest 0.5 cm using by tape meter fixed to a wall. Body mass index (BMI) was calculated by dividing weight (kg) by the square of height (meter). Furthermore, waist circumference (at the level midway between the lowest rib margin and the iliac) and hip circumference (at the widest point over the buttocks) were measured to the nearest 0.5 cm using a non-stretchable tape measure. Subsequently, the waist-hip ratio (WHR) was calculated.

### Statistical analysis

The normal distribution of variables between case and control groups was assessed using a histogram chart and the Kolmogorov-Smirnoff test. The mean values of continuous and categorical variables were compared using the independent sample t-test or Mann-Whitney U (for non-normal variables) and the chi-square test.

The correlation coefficient (r) between different insulin indices was calculated using a partial correlation test. Each insulin indices were categorized as tertiles based on the three equal categories among controls. The odds ratios (ORs) and 95% confidence intervals (95% CIs) of breast cancer across tertiles of insulin indices were calculated by logistic regression analysis adjusted for various potential confounders in different models.

For selecting the confounding variables, we conducted a univariate test for the list of variables discussed in previous studies and selected those with P-values lower than 0.2. So the final model was adjusted for age, age at first pregnancy, family history of cancer, menopausal status, anti-inflammatory drugs, vitamin D supplement, BMI (for EDIH and EDIR), and physical activity (for EDIH and EDIR).

After testing for interaction, analyses were stratified by menopausal status, cancer family history, and OCP use (P-interaction<0.05). Statistical tests were performed using SPSS software (v.16.0). *P*-values < 0.05 were considered to be statistically significant.

## Results

The baseline characteristics of participants, including demographic and lifestyle variables, medical history, and dietary intakes, are indicated in Table 1. Participants’ mean ± SD of age and BMI were 47.9 ± 10.3 years and 29.4 ± 5.5 kg/m2, respectively. Individuals in the case group had higher age, first pregnancy age, postmenopausal women percent, and cancer family history, whereas they had lower anti-inflammatory drug consumption and vitamin D supplement intake than the control group (*P*<0.05). There were no significant differences between cases and controls in insulinemic indices, including EDIH, EDIR, ELIH, EDIH, and other variables.

**Table 1:** Characteristics of breast cancer cases and controls at Imam Hossain and Shohada hospitals, Tehran (Iran) between September 2015 and February 2016

Variables	Cases(*n*=134)*	Controls(*n*=267)*	*P*-value^†^
Age(year)^a^	49.4 ± 10.6	47.1 ± 10.1	0.03
Menarche age(year)	13.6 ± 1.6	13.6 ± 1.6	NS
Marriage age(year)	19.4 ± 6.6	18.3 ± 5.7	NS
First pregnancy age(year)	19.6 ± 8.6	18.2 ± 7.4	0.04
BMI (kg/m^2^)	30.1 ± 5.7	29.1 ± 5.9	NS
WHR^a^	0.9 ± 0.1	0.9 ± 0.1	NS
Physical activity (MET-h/day)	32.9 ± 5.4	32.7 ± 5.2	NS
Smoking (yes), n (%)	4 (3.0)	9 (3.4)	NS
Education (diploma and higher), n (%)	62 (46.3)	108 (40.4)	NS
Occupation (yes), n (%)	109 (81.3)	211 (79)	NS
Breastfeeding time(*month*)	40.9 (38.3)	47.3 (40.9)	NS
Menopausal status, n (%)			0.04
pre-menopause	62 (46.3)	153 (57.3)	
post-menopause	72 (53.7)	114 (42.7)	
Marital status: n (%)			NS
Single	9 (6.8%)	16 (6.0%)	
Married	105 (78.9%)	206 (77.4%)	
Divorced	5 (3.8%)	13 (4.9%)	
Widowed	14 (10.5%)	31 (11.7%)	
**Medical history**			
Breast cancer family history (YES): n (%)	11 (8.2)	12 (4.5)	NS
Cancer family history (YES): n (%)	41 (30.6)	55 (20.7)	0.03
Benign breast diseases history (YES): n (%)	12 (9.0)	14 (5.3)	NS
Inflammatory disease history (YES): n (%)	15 (10.4)	35 (13.2)	NS
Abortion history (YES): n (%)	52 (38.8)	78 (29.2)	NS
OCP use, n (%)	67 (50.0)	149 (55.8)	NS
Anti-inflammatory drugs (YES): n (%)	10 (7.5)	46 (17.3)	0.01
HRT (YES): n (%)	7 (5.2)	29 (10.9)	NS
**Dietary intakes**			
Daily energy intake(kcal/day)	2562 ± 612	2753 ± 798	NS
EDIH	0.25 ± 0.19	0.23 ± 0.22	NS
EDIR	0.88 ± 0.31	0.94 ± 0.41	NS
ELIH	1.53 ± 0.33	1.47 ± 0.31	NS
ELIR	5.61 ± 1.77	5.96 ± 2.52	NS
Vitamin D supplement (YES): n (%)	20(4.9)	65(24.4)	0.03
Calcium supplement (YES): n (%)	35 (26.1)	73 (27.3)	NS
Iron supplement (YES): n (%)	20 (14.9)	45 (16.9)	NS
Folic acid supplement (YES): n (%)	16 (11.9)	30 (11.2)	NS
Omega-3 supplement (YES): n (%)	8 (6.0)	31 (11.6)	NS
Herbal drug (YES): n (%)	26 (19.4)	72 (27.1)	NS

*NS* non-significant

^a^Normal distribution

^*^Data presented as mean ± SD and number (percent) for quantitative and qualitative variables, respectively

^†^Student t-test or Mann-Whitney was used for continuous variables, and the Chi-square test was used for categorical variables

Table 2 shows the correlation coefficient of insulin indices. There was a significant correlation between EDIH and EDIR(*r*=0.263), EDIH and ELIH(*r*=0.215), and EDIR and ELIR(*r*=0.851).

**Table 2:** The correlation coefficients of insulin indices

	**EDIH**	**EDIR**	**ELIH**	**ELIR**
**EDIH**	1	0.263*	0.215*	0.091
**EDIR**	0.263*	1	0.048	0.851*
**ELIH**	0.215*	0.048	1	0.094
**ELIR**	0.091	0.851*	0.094	1

^*^<0.001

The association of insulin indices with the risk of BC is presented in Table 3. A significant positive association was observed between higher EDIH score and risk of BC in the highest compared to the lowest tertiles in the age and age first pregnancy model (OR: 2.85; 95% CI: 1.05 – 3.23, *P* for trend=0.059). After adjusting for confounding factors in the final model, participants with the highest EDIH score had higher odds of BC than those with the lowest EDIH score (OR: 2.24; 95% CI: 1.21 – 4.12, *P* for trend=0.016). However, based on all logistic regression models, there is no significant association between ELIH, EDIR, and ELIR and the risk of BC.

**Table 3:** The association between insulinemic indices and breast cancer in adult women in Imam Hossain and Shohada hospitals, Tehran between September 2015 and February 2016

Variable	Tertiles of the insulinemic indices			
	1	2	3	*P*-trend
**EDIH**				
EDIH value cut points	<0.128	0.128 to 0.259	>0.259	
Median score	0.064	0.198	0.373	
cancer /control	28/88	56/89	49/88	
Crude	Ref (1.00)	1.95 (1.14 – 3.36)	1.73 (1.00 – 3.00)	0.094
Model 1^a^	Ref (1.00)	2.00 (1.16 – 3.47)	2.85 (1.05 – 3.23)	0.059
Model 2^b^	Ref (1.00)	1.80 (1.03 – 3.17)	1.81 (1.02 – 3.23)	0.068
Model 3^c^	Ref (1.00)	2.00 (1.12 – 3.6)	2.24 (1.21 – 4.12)	0.016
**EDIR**				
EDIR value cut points	<0.733	0.733 to 1.044	>1.044	
Median score	0.578	0.867	1.288	
cancer /control	51/88	44/89	38/88	
Crude	Ref (1.00)	0.84 (0.51 – 1.39)	0.74 (0.44 – 1.25)	0.265
Model 1^a^	Ref (1.00)	0.86 (0.52 – 1.43)	0.74 (0.44 – 1.25)	0.259
Model 2^b^	Ref (1.00)	0.87 (0.52 – 1.46)	0.76 (0.44 – 1.30)	0.318
Model 3^c^	Ref (1.00)	0.90 (0.57 – 1.54)	0.94 (0.51 – 1.74)	0.846
**ELIH**				
ELIH value cut points	<1.302	1.302 to 1.586	>1.586	
Median score	1.170	1.452	1.786	
cancer /control	33/87	53/87	47/86	
Crude	Ref (1.00)	1.58 (0.93 – 2.68)	1.46 (0.85 – 2.49)	0.206
Model 1^a^	Ref (1.00)	1.50 (0.88 – 2.55)	1.40 (0.80 – 2.39)	0.287
Model 2^b^	Ref (1.00)	1.56 (0.90 – 2.71)	1.49 (0.85 – 2.61)	0.202
Model 3^d^	Ref (1.00)	1.50 (0.86 – 2.62)	1.43 (0.81 – 2.53)	0.255
**ELIR**				
ELIR value cut points	<4.543	4.543 to 6.486	>6.486	
Median score	3.882	5.420	7.761	
cancer /control	39/87	57/88	37/87	
Crude	Ref (1.00)	1.42 (0.86 – 2.35)	0.96 (0.56 – 1.45)	0.732
Model 1^a^	Ref (1.00)	1.41 (0.85 – 2.34)	0.97 (0.56 – 1.67)	0.754
Model 2^b^	Ref (1.00)	1.48 (0.88 – 2.51)	1.03 (0.58 – 1.81)	0.913
Model 3^d^	Ref (1.00)	1.65 (0.96 – 2.83)	1.29 (0.69 – 2.38)	0.527

^a^Model 1: adjusted for age and age at first pregnancy

^b^Model 2: adjusted for model 1 and family history of cancer (yes, no), menopausal status (yes, no), anti-inflammatory drugs (yes, no), and vitamin D supplement (yes, no)

^c^Model 3: adjusted for model 2 and energy, BMI, physical activity, and smoking

^d^Model 3 adjusted for model 2 and energy and smoking

Table 4 showed the adjusted OR (95% CI) of BC per one SD increment of insulin indices in subgroup analysis based on three variables, including menopausal status, cancer family history, and OCP use. Each SD increase in the EDIH score was associated with an increased risk of BC among postmenopausal women (OR: 1.74, 95% CI: 1.13–2.69) and those who do not use OCP (OR: 1.44;95% CI:1.02 – 2.04). Also, each SD increment of the ELIH score was related to a higher OR of BC based on postmenopausal status (OR; 1.98; 95% CI: 1.35 – 2.89), family history of cancer (OR: 1.94; 95% CI: 1.10 – 3.42), and no use of OCP (OR: 1.46; 95% CI: 1.00 – 2.12). The dietary or lifestyle indices for insulin resistance (EDIR and ELIR) showed no association with BC odds among subgroups.

**Table 4:** The association between per 1-SD increment of each insulinemic indices and breast cancer in different subgroups in adult women in Imam Hossain and Shohada hospitals, Tehran (Iran) between September 2015 and February 2016

**Subgroups**	**EDIH**		**EDIR**		**ELIH**		**ELIR**	
	**OR**^a^**(95% CI)**	**P-value**	**OR**^a^**(95% CI)**	**P-value**	**OR**^b^**(95% CI)**	**P-value**	**OR**^b^**(95% CI)**	**P-value**
**Menopausal status**								
** Pre-menopause**	1.11 (0.82 – 1.51)	0.496	0.83 (0.58 – 1.21)	0.340	0.91 (0.67 – 2.83)	0.536	0.86 (0.60 – 1.25)	0.428
** Post-menopause**	1.74 (1.13 – 2.69)	**0.013**	1.06 (0.71 – 1.58)	0.794	1.98 (1.35 – 2.89)	**<0.001**	1.09 (0.74 – 1.62)	0.664
**Cancer family history**								
** No**	1.18 (0.89 – 1.56)	0.253	0.902 (0.66 – 1.23)	0.520	1.19 (0.87 – 1.46)	0.359	1.00 (0.73 – 1.37)	0.998
** Yes**	1.56 (0.91 – 9.67)	0.103	0.93 (0.51 – 1.70)	0.820	1.94 (1.10 – 3.42)	**0.021**	0.78 (0.45 – 1.35)	0.368
**OCP use**								
** No**	1.44 (1.02 – 2.04)	**0.038**	1.23 (0.74 – 1.73)	0.580	1.46 (1.00 – 2.12)	**0.048**	1.11 (0.73 – 1.69)	0.619
** Yes**	1.16 (0.80 – 1.67)	0.438	0.81 (0.56 – 1.16)	0.254	1.15 (0.85 – 1.56)	0.358	0.90 (0.63 – 1.29)	0.56

^a^Adjusted for age and age at first pregnancy, family history of cancer (yes, no), menopausal status (yes, no), anti-inflammatory drugs (yes, no), vitamin D supplement (yes, no), energy, BMI, Physical activity, and smoking

^b^Adjusted for age and age at first pregnancy, family history of cancer (yes, no), menopausal status (yes, no), anti-inflammatory drugs (yes, no), vitamin D supplement (yes, no), energy, and smoking

## Discussion

Current research provides the first evidence about the association between EDIH, EDIR, ELIH, and ELIR and the risk of BC. Based on our findings, EDIH was related to the higher risk of BC based on fully adjusted models. Furthermore, elevated EDIH level was associated with higher BC risk based on postmenopausal and non-using OCP based on subgroup analysis. Moreover, ELIH increment in postmenopausal women, those with a family history of cancer, and not using OCP was associated with an increased risk of BC.

Our findings are consistent with some previous studies investigating the possible association of the insulinemic potential of diet and lifestyle with the risk of various cancers. There is evidence that the higher score of EDIH was associated with a 26% increased colorectal cancer risk in men and women [18]. Similarly, Yang et al. have observed a positive association between higher scores of EDIR and increased risk of hepatocellular carcinoma [19]. Another study has reported that EDIH and ELIH were significantly associated with digestive tract cancers [20]. Furthermore, two studies claimed that participants with hyperinsulinemic diets had a greater risk of advanced and fatal prostate cancer [9, 21]. Although EDIR was associated with increased multiple myeloma risk in another study, EDIH did not show any significant relationship with the risk of multiple myeloma [13]. Although Cheng et al. declared that a higher score of EDIH is potentially related to colon cancer risk, no significant association was observed between a potential insulinemic diet and the risk of colon cancer recurrence, survival, or mortality in patients with late-stage (III) colon cancer [22]. EDIH, EDIR, ELIH, and ELIR are indicators that have been implicated in the pathogenesis of various cancer and complications [23, 24]. Insulin is a key regulator hormone in cell growth and energy metabolism. Up-regulation of insulin secretion and insulin resistance results in elevated IGF-1 production and bioavailability, resulting in cell proliferation and tumor growth [25]. It should be noted that dietary insulin indices are related to insulin resistance [26]. Moreover, some evidence shows that the dietary hyperinsulinemia index potentially affects cancer progression, especially in those with a low level of PA [18].

The subgroup analyses have revealed a positive association between EDIH and ELIH scores and the risk of BC in women who did not use OCP. Previous studies demonstrated that OCP use might increase BC’s risk [27]; however, this increased risk is slight and highly depends on different underlying factors like age, genetics, duration of use, and formulation of pills [28–31]. Besides, most of those studies included women who used OCP in the 1980s or earlier, when the pills tended to have a higher hormone content than they do now [32]. Furthermore, according to previous studies, OCP consumption increases insulin resistance risk [33, 34]. It is possible that in patients without a history of OCP use, due to fewer metabolic disorders than individuals with this history, the role of hyperinsulinemic diet and lifestyle in the development of metabolic disorders and increased cancer risk for each SD increase in scores is more prominent and therefore the overall relationship is significant.

A higher EDIH and ELIH score also increased the risk of BC in postmenopausal women. Increased endogenous estrogen status [35] is associated with postmenopausal breast cancer risk [36]. Although insufficient evidence is available on the possible effect of pre-and post-menopausal cancer, the risk of uterine, ovarian, and breast cancers increases with aging, especially after age 55 years old [37]. Lifestyle changes, anthropometric changes, including elevated BMI and adiposity, low physical activity, and hormone replacement therapy, increase by getting older and in those experiencing a postmenopausal period, can enhance BC risk [38, 39]. For instance, Toklu and Nogay indicate that unhealthy dietary patterns and eating deep-fried red meat, a sedentary lifestyle, and a high BMI, especially during the postmenopausal period, are risk factors for BC [40].

In addition, according to subgroup analysis results, a significant direct association of ELIH with the risk of BC was observed in subjects with a history of cancer. There is some evidence on the relationship between family history and the risk of BC, which expresses that first-degree family history can promote invasive BC risk [41]. For instance, Reiner et al. reported that having a first-degree relative with BC is associated with higher BC risk [42]. Also, Ahern et al. demonstrated that first- and second-degree relatives can estimate BC occurrence [43]. As well as hereditary has an important role in breast cancer incidence [44], family members may have a similar unhealthy lifestyle and dietary behaviors.

Strengths of the current study include the novelty of the investigation and data gathering from well-documented research centers. Furthermore, we have used valid and reliable questionnaires for dietary intake and physical activity evaluation. In addition, multiple logistic regression models, considering various confounders, were used in our study. However, we acknowledge some limitations.

The main limitation of the current study was the case-control study design which cannot provide a causal relationship. Also, our trained dietitians were not masked about cases and controls; however, during training, focus was given to minimize possible information bias to collect the data without mental background about participants’ cancer status. Since the study is conducted in Tehran, Iran, we cannot generalize the results to the other women population. Also, recall bias is possible due to using FFQ for dietary assessment. Furthermore, conducting stratified analyses based on three variables may increase the possibility of showing chance findings. However, the findings about EDIH, EDIR, and ELIR were repeated. Only ELIH showed a positive association with cancer among postmenopausal women, those with the cancer family history, and those with no OCP consumption. So, these findings should be tested among mentioned subgroups in other studies for a better perception of the ELIH-cancer relationship.

## Conclusion

In conclusion, our results showed a possible link between EDIH and higher BC risk. Also, higher EDIH and ELIH scores were associated with a higher risk of BC in women in the postmenopausal period, having a family history of BC, and those who do not use OCP.

## Data Availability

The data analyzed in the present study are available by the corresponding author on a reasonable request.

## Abbreviations

BC: Breast cancer
EDIH: Empirical dietary index for hyperinsulinemia
ELIH: Empirical lifestyle index for hyperinsulinemia
EDIR: Empirical dietary index for insulin resistance
ELIR: Empirical lifestyle index for insulin resistance
OR: Odds ratio
CI: Confidence interval
SD: Standard deviation
OCP: Oral contraceptive pill
IGF-1: Insulin-like growth factor-1
IR: Insulin resistance
C-peptide: Connecting peptide
TG: Triglyceride
HDL-C: High-density lipoprotein-cholesterol
PA: Physical activity
BMI: Body mass index
HCC: Hepatocellular carcinoma
CRC: Colorectal cancer
MM: Multiple myeloma
FFQ: Food frequency questionnaire
USDA: United States Department of Agriculture
SPSS: Statistical Package for the Social Sciences
Kg: Kilogram
